# Metabolomic signature of exposure and response to citalopram/escitalopram in depressed outpatients

**DOI:** 10.1038/s41398-019-0507-5

**Published:** 2019-07-04

**Authors:** Sudeepa Bhattacharyya, Ahmed T. Ahmed, Matthias Arnold, Duan Liu, Chunqiao Luo, Hongjie Zhu, Siamak Mahmoudiandehkordi, Drew Neavin, Gregory Louie, Boadie W. Dunlop, Mark A. Frye, Liewei Wang, Richard M. Weinshilboum, Ranga R. Krishnan, A. John Rush, Rima Kaddurah-Daouk

**Affiliations:** 1 Department of Biomedical Informatics, University of Arkansas for Medical Sciences, Little Rock, AR USA; 2 Department of Psychiatry and Psychology, Mayo Clinic, Rochester, MN USA; 3 Department of Psychiatry and Behavioral Sciences, Duke University School of Medicine, Durham, Durham, NC USA; 4 Institute of Bioinformatics and Systems Biology, Helmholtz Zentrum München – German Research Center for Environmental Health, Neuherberg, Germany; 5 Department of Molecular Pharmacology & Experimental Therapeutics, Mayo Clinic, Rochester, MN USA; 6 Sanofi, Bridgewater, NJ USA; 7 Department of Psychiatry and Behavioral Sciences, Emory University School of Medicine, Atlanta, GA USA; 8 Department of Psychiatry, Rush University Medical Center, Chicago, IL USA; 9 Texas Tech University, Health Sciences Center, Permian Basin, Odessa, TX USA; 10 Duke-National University of Singapore, Singapore, Singapore; 11 Department of Medicine, Duke University, Durham, NC USA; 12 Duke Institute of Brain Sciences, Duke University, Durham, NC USA

**Keywords:** Depression, Scientific community

## Abstract

Metabolomics provides valuable tools for the study of drug effects, unraveling the mechanism of action and variation in response due to treatment. In this study we used electrochemistry-based targeted metabolomics to gain insights into the mechanisms of action of escitalopram/citalopram focusing on a set of 31 metabolites from neurotransmitter-related pathways. Overall, 290 unipolar patients with major depressive disorder were profiled at baseline, after 4 and 8 weeks of drug treatment. The 17-item Hamilton Depression Rating Scale (HRSD_17_) scores gauged depressive symptom severity. More significant metabolic changes were found after 8 weeks than 4 weeks post baseline. *Within the tryptophan pathway*, we noted significant reductions in serotonin (5HT) and increases in indoles that are known to be influenced by human gut microbial cometabolism. 5HT, 5-hydroxyindoleacetate (5HIAA), and the ratio of 5HIAA/5HT showed significant correlations to temporal changes in HRSD_17_ scores. *In the tyrosine pathway*, changes were observed in the end products of the catecholamines, 3-methoxy-4-hydroxyphenylethyleneglycol and vinylmandelic acid. Furthermore, two phenolic acids, 4-hydroxyphenylacetic acid and 4-hydroxybenzoic acid, produced through noncanconical pathways, were increased with drug exposure. *In the purine pathway*, significant reductions in hypoxanthine and xanthine levels were observed. Examination of metabolite interactions through differential partial correlation networks revealed changes in guanosine–homogentisic acid and methionine–tyrosine interactions associated with HRSD_17_. Genetic association studies using the ratios of these interacting pairs of metabolites highlighted two genetic loci harboring genes previously linked to depression, neurotransmission, or neurodegeneration. Overall, exposure to escitalopram/citalopram results in shifts in metabolism through noncanonical pathways, which suggest possible roles for the gut microbiome, oxidative stress, and inflammation-related mechanisms.

## Introduction

Major depressive disorder (MDD) is a common, often disabling condition affecting over 300 million individuals worldwide^1^. Selective serotonin reuptake inhibitors (SSRIs) are common first-line treatments for MDD^2,3^. They are believed to increase the extracellular availability of the neurotransmitter serotonin by limiting its reabsorption into the presynaptic cell, so that serotonin levels are increased in the synaptic cleft and available for binding to postsynaptic receptors. Responses to antidepressant medications are modest. Only about half the patients respond to the first medication; only one in three achieves symptom remission, which is the virtual absence of symptoms and the aim of treatment^4^_._ Some patients do well on a single medication, while others require medication combinations or alternative interventions. Clinical symptoms are insufficient to guide appropriate treatment selection^5^ and, presently, treatments are therefore selected empirically relying on a “trial and error” approach^6,7^.

Metabolomics, a promising new approach to understanding depression and other neuropsychiatric disorders^8–11^, could help inform treatment selection^12,13^. Metabolomic profiles provide informative readouts on pathways and biological networks implicated in various diseases or their treatments. Metabolomic signatures have been identified for several psychiatric disorders, such as MDD^14^, bipolar disorder^15,16^, and schizophrenia^17–19^. Most studies of mood disorders have implicated tryptophan (TRP), tyrosine, and purine metabolism, since historically, neurotransmission and serotonergic signaling were key focus areas of investigation^14^. The TRP pathway along with its three branches of metabolism to serotonin/melatonin/5-hydroxyindoleacetate, kynurenine (KYN), and indole derivatives, seems to be affected in the depressed state^20–28^. The purine pathway, whose regulation seems to be connected to TRP metabolism, has also been implicated in depression and other psychiatric disorders^29^. Among patients in remission from a major depressive episode, a metabolomic signature that included methionine, glutathione along with metabolites in the purine and TRP pathways, has been identified^30^.

Pharmacometabolomics has also revealed that patients’ metabolomic profiles (metabotypes), both prior to and early during treatment, can inform treatment outcomes^10,31^. This approach has been applied to antihypertensive^32^ and antiplatelet^33^ therapies. We have used this approach to predict treatment outcomes and to identify specific metabolomic pathways that were changed in response to sertraline^34,35^ and to ketamine^36^, a promising agent for treatment-resistant depression. We have also employed a “pharmacometabolomics-informed pharmacogenomics” research strategy^11^ to investigate the role of genetics in response to citalopram or escitalopram^37,38^, thereby advancing the goal of precision medicine for depression^31^. However, the acute and longer-term effects of treatment with citalopram or escitalopram on pathways, critical to the pathobiology or pharmacotherapy of depression, and the relationship to clinical outcomes have not been reported.

This report used metabolomic analyses with selected metabolites in the tryptophan, tyrosine, purine, tocopherol, and the related pathways in a sample of nonpsychotic depressed outpatients who were treated for 8 weeks with citalopram or escitalopram to address the following questions:

- The metabolomic signature of exposure to escitalopram/citalopram: which metabolite changes occurred from baseline to week 4, and from baseline to week 8 of treatment?
- The metabolomic signature of response: which metabolomic changes were related to changes in depressive symptoms (HRSD_-17_), longitudinally, in the overall population and also in responders versus nonresponders?
- The interrelationships between metabolites: what are the relationships among metabolites, both within and between pathways, before and after treatment with the drug?

## Methods

### Study design and participants

We used samples from the Mayo Clinic NIH-Pharmacogenomics Research Network-Antidepressant Pharmacogenomics Medication Study (PGRN-AMPS) which recruited a total of 803 MDD patients^39^. Patient selection, symptomatic evaluation, and blood sample collection for the PGRN-AMPS clinical trial have been described elsewhere^24,38–40^. Briefly, MDD patients were required to have a baseline HRSD_17_ score ≥ 14, and all patients who completed 8 weeks of treatment (*n* = 290) were treated with one of the two SSRIs, citalopram or escitalopram. Depressive symptoms were assessed with HRSD_17_ at baseline, week 4, and week 8 of SSRI treatment. Blood samples were collected at these same time points.

The HRSD_17_ was used to ascribe “response”—defined as at least 50% reduction in the total score from baseline to exit; “remission” —an exit HRSD_17_ score of 7 or less; and “complete-non-response”—less than 30% reduction in the HRSD_17_ total score from baseline to exit^39^. Genome-wide association studies for plasma concentrations of the SSRIs and metabolite levels^40^ and for response^41^ in this trial have been published previously. The trial was designed as a parallel to the large National Institute of Mental Health—funded “the Sequenced Treatment Alternatives to Relieve Depression” (STAR*D) clinical trial^42^ for the purpose of replication of the identified genetic markers.

### Metabolomic profiling

A targeted, liquid chromatography–electrochemical coulometric array (LCECA) metabolomics platform^43^ was used to assay metabolites in plasma samples from the three time points, baseline, 4 weeks, and 8 weeks. This platform was used to identify and quantify 31 neurotransmitter-related metabolites (against standards) primarily from the TRP, tyrosine, and tocopherol pathways, including serotonin. A list of the metabolites that were quantitatively measured using this platform is presented in Table 1.

**Table 1:** List of metabolites and pathways analyzed in the study

Metabolite by pathways	Abbreviation	Metabolite by pathways	Abbreviation
**Tryptophan**		**Phenylalanine/tyrosine**	
3-Hydroxykynurenine	3OHKY	4-hydroxybenzoic acid	4HBAC
5-Hydroxyindoleacetic acid	5HIAA		
5-Hydroxytryptophan	5HTP	**Purine**	
Indole-3-acetic acid	I3AA	Guanine	G
Kynurenine	KYN	Guanosine	GR
Serotonin	5HT	Hypoxanthine	HX
Tryptophan	TRP	Uric acid	URIC
		Xanthine	XAN
**Tyrosine**		Paraxanthine	PXAN
4-Hydroxyphenylacetic acid	4HPAC	Xanthosine	XANTH
4-Hydroxyphenyllacetic acid	4HPLA		
Homogentisic acid	HGA	**1 Carbon** **+** **GSH**	
Homovanillic acid	HVA	Methionine	MET
Methoxy-hydroxyphenyl glycol	MHPG	Cysteine	CYS
Tyrosine	TYR		
Vanillylmandelic acid	VMA	**Other**	
		Salicylate	SA
**Tocopherol**		Alpha-Methyltryptophan	AMTRP
Tocopherol-alpha	ATOCO	Indole-3-propionic acid	I3PA
Tocopherol-delta	DTOCO	Theophylline	Theophylline
Tocopherol-gamma	GTOCO		

#### Analysis method

The long-gradient LCECA method used for this analysis can resolve compounds at picogram levels through electrochemical detection (resulting from oxidation or reduction reactions) including multiple markers of oxidative stress and protection. This method utilizes a 120-min gradient from (0%) organic modifier with an ion-pairing agent (i.e., pentane sulfonic acid) to a highly organic mobile phase with methanol (80%)/isopropanol (10%)/acetonitrile (10%). An array of 16 serial coulometric electrochemical detectors is set at incremental potentials from 0 to 900 mV, responding to oxidizable compounds such as tocopherol in lower potential sensors and higher oxidation potential compounds such as hypoxanthine in the higher potential channels.

#### Analysis sequence and data output

At the time of preparation, a pool was created from small aliquots of each sample in the study, which was then treated identically to a sample. All of these assays were executed in sequences that included mixed standard, five samples, pool, five samples, mixed standard, and so on and so forth. In this study, all sample run orders were randomized. The sequences decreased possible analytical artifacts during further data processing. Data were time normalized to a pool at the midpoint of the study, aligning major peaks to 0.5 s and minor peaks to 0.5–2 s. Details on the LCECA methods are described in previously published work^35,41,44–50^.

### Data analysis

All data preprocessing and analysis were performed with R (version 3.4.2) and Bioconductor (version 3.3) statistical packages.

#### Preprocessing

This study’s data extraction protocol followed the STORBE guidelines^46^. All metabolite data were first checked for missing values (none were detected at >20% missing abundances) and were subjected to imputation by the *k*-nearest neighbor algorithm^51^. Data were then log2 transformed and scaled to unit variance prior to statistical analyses.

#### Univariate analysis

To define the effect of drug exposure over 4 weeks and 8 weeks of treatment, linear mixed effects models (using the R package nlme^52^) were fitted on each metabolite adjusting for age, gender, and HRSD_17_ scores at baseline with subjects as random variable. Analyses were conducted separately for 4 and 8 weeks. Linear mixed effects models were also used to determine associations between the changes in metabolites and changes in HRSD_17_ over time, with age and gender as covariates, and using subjects as random variable. All *p*-values were used to calculate the false discovery rates by Benjamini–Hochberg method^53^, and a cutoff point of 10% was used. A two-step regression strategy was used to find metabolites with significant temporal changes and significant differences between responders and nonresponders using the maSigPro library in R^54^. First, a least-squared technique was employed to identify differential metabolites in a global regression model, using dummy variables for experimental groups. Second, stepwise regression was applied to select variables that differed between the experimental groups and find significantly different metabolite profiles between the groups.

#### Partial correlation networks with cluster subgraph analysis

The relationship between metabolites in a complex disease setting can be represented in terms of partial correlation networks, where each node represents a metabolite and each edge between two metabolites represents that two variables are not independent after conditioning on all variables in the dataset. These edges have a weight, edge weights, which are the partial correlation coefficients. Here, we estimated the partial correlation matrix for all of the metabolites using the least absolute shrinkage and selection parameter (LASSO) to obtain the sparse inverse covariance matrix to avoid overfitting and spurious correlations. Thus, it can be reasonably expected that the regularized partial correlation networks will provide accurate estimates of the underlying relationships between the metabolites in metabolic pathways and reactions. The LASSO regularization parameter was set via EBIC or Extended Bayesian Information criterion^50^. Finally, the walktrap algorithm, which is based on random walks to capture cluster structures in a network, is used to identify clusters of strongly interacting metabolites^45^. The final network with cluster subgraphs is formed by the median pairwise partial correlations over 1000 bootstrap estimations and plotted using the Fruchterman–Reingold layout. We further included the HRSD_17_ scores in our partial correlation network models to perform differential network analysis. The overall statistical impact of HRSD_17_ scores on the metabolite interactions was calculated based on measuring structure invariance between two networks, high HRSD_17_ and low HRSD_17_ networks, constructed using a median split of the variable. Permutation tests were used to determine the significance of structure and edge invariances between the two networks^55^. The metabolite–metabolite partial correlations that were of differential strength between networks of high and low HRSD_17_ networks were further validated for significant interaction effects through linear regression analysis.

#### Candidate metabolic trait GWAS with HGA/GR and MET/TYR ratios

For 288 of the 290 subjects in this study we had genotype data for the Illumina human 610-Quad BeadChips (Illumina, San Diego, CA, USA) available, as described previously^38,41^. Genotype QC using PLINK and imputation followed standard protocols. Briefly, raw genotype data were filtered for variants with call rate <5%, minor allele frequency (MAF) <5%, and Hardy–Weinberg equilibrium HWE *p* < 1 × 10^−5^^49^. The data was then subjected to prephasing using SHAPEIT2 (ver. 2.12)^48^, followed by imputation with IMPUTE2 (ver. 2.3.2)^47^ using 1000 genomes phase 3 version 5^56^ haplotypes as a reference. Post-imputation QC included filtering variants for IMPUTE info score < 0.5, call rate and MAF < 5%, and HWE *p* < 1 × 10^−5^, resulting in a final set of 5.55 mio SNPs with 99.14% genotyping rate. To remove any potential for spurious associations due to population stratification, we used a set of about 100,000 SNPs pruned for the LD structure and retrieved the first five principal component eigenvectors (PCs). Metabolite data for the HGA/GR and Met/TYR ratios were log transformed, centered to zero mean, and scaled to unit variance. In addition, for candidate GWAS, we excluded values that were more than 4 standard deviations from the mean. We then performed GWAS for HGA/GR and MET/TYR at each time point while adjusting for age, sex, and PCs 1–5. We reran the GWAS additionally adjusting for HDRS_17_ scores at each time point to eliminate the effects linked to depression severity.

## Results

### Patient characteristics

Plasma metabolite data were available from 290 MDD patients. The average age of the patient cohort was 39.8 (±13.1) years. Females comprised of 66% of the study cohort, while males were at 34%. The response rate to the drug, based on HRSD_17_ scores, was 69.3% after 8 weeks, compared with 30.7% who were classified as nonresponders for this study. The depressive status of the patients, as determined by the HRSD_17_ scores, decreased over time with the drug treatment, from an average of 21.9 (±4.9) at baseline to 11.6 (±6.4) at week 4 and 8.6 (±5.5) at week 8. Demographic and clinical characteristics are detailed in Supplemental Table 1.

### Metabolite changes at weeks 4 and 8 compared with baseline, in response to the drug

**Fig. 1:**
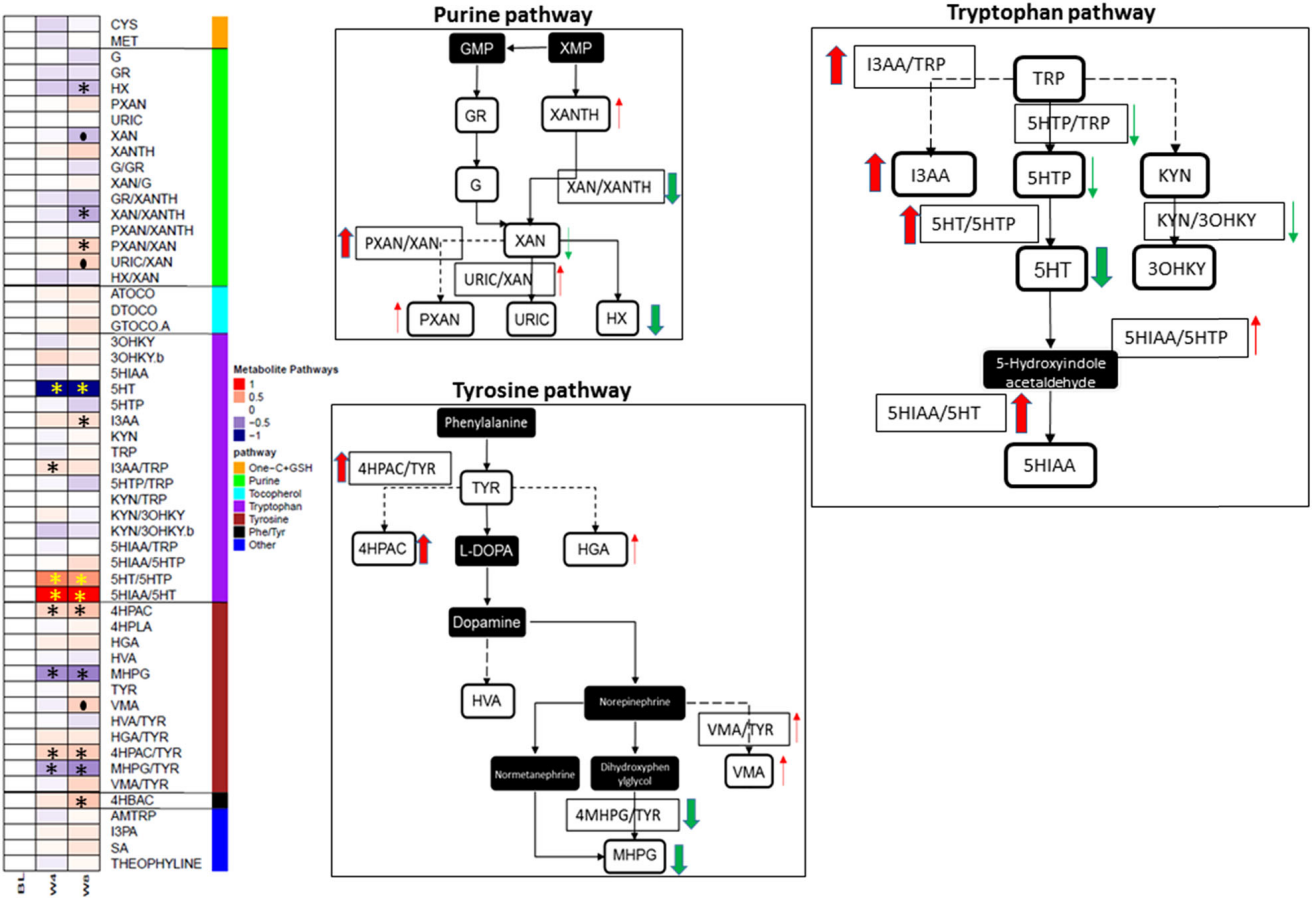
Metabolic signature of drug exposure. **a** Shows the heatmap of metabolite changes at baseline, week 4, and week 8, normalized to baseline levels. **b**–**d** Show changes within the purine, tryptophan, and tyrosine pathways

Several metabolites in the purine, tryptophan, and tyrosine pathways changed, following 4 weeks of drug therapy. However, perturbations in the metabolite levels were in general, greater and more significant after 8 weeks of treatment (Supplemental Table 2). Figure 1 illustrates changes within key pathways evaluated after 8 weeks of treatment.

#### Tryptophan pathway

Dramatic changes were observed in serotonin (5HT) and the ratio 5HIAA/5HT, both at week 4 and week 8. At both time points, 5HT showed substantial decreases and the 5HIAA/5HT ratio was significantly elevated. While TRP itself did not show a notable change, its indole-containing metabolite I3AA was significantly elevated, as was the ratio of I3AA/TRP, possibly indicating a shift away from the serotonergic pathway of TRP metabolism. Interestingly, another indole-containing compound that is known to be produced only by gut microbiota in humans, I3PA, was also increased at 8 weeks (unadjusted *p*-value < 0.02). No statistically significant alterations were observed in the KYN branch of TRP metabolism.

#### Tyrosine pathway

A similar trend of a shift to noncanonical branches of tyrosine metabolism was also observed in this pathway. MHPG, the major metabolite of the neurotransmitter norepinephrine and the ratio MHPG/TYR showed significant reductions in their blood levels at both 4 and 8 weeks while VMA, a norepinephrine end metabolite, showed significant elevations at 8 weeks compared with baseline. A phenolic acid, 4HPAC, and its ratio to TYR (4HPAC/TYR) were significantly increased at both 4 and 8 weeks. Another phenolic derivative from the phenylalanine/tyrosine pathway, 4-hydroxybenzoic acid (4HBAC), was also significantly elevated at 8 weeks.

#### Purine pathway

The purine metabolites HX and XAN and the ratio XAN/XANTH were decreased significantly, while the ratios PXAN/XAN and URIC/XAN were elevated at 8 weeks compared with baseline, indicating a similar decline in the canonical pathway of purine metabolism, as observed in the tryptophan and tyrosine pathways.

Other metabolites that showed significant changes, albeit at unadjusted *p* values <0.05, were the purine metabolites, G, PXAN, and XANTH; the TRP metabolite, 5HTP; the tyrosine metabolite, HGA; and other metabolites, such as salicylic acid (SA).

### Metabolomic changes associated with changes in depressive symptoms (HRSD_17_)

**Fig. 2:**
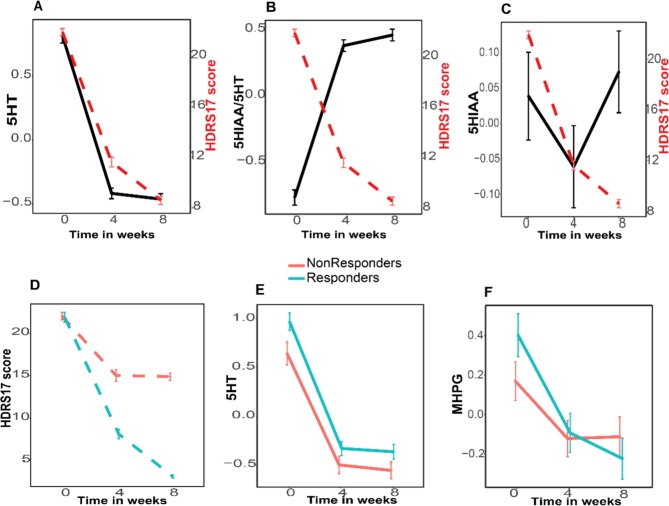
Metabolite changes associated with HRSD_17_ scores. **a** 5HT (Serotonin), **b** 5HIAA/5HT ratio, and **c** 5HIAA (5-hydroxyindoleacetic acid). Temporal changes in (**d**) HRSD_17_ scores, (**e**) 5HT, and (**f**) MHPG differed significantly between responders and nonresponders. The error bars represent standard error of the mean

Using linear mixed models, we examined the association between temporal changes in metabolite levels (across three time points, baseline, 4 weeks, and 8 weeks) and the temporal changes in patients’ HRSD_17_ scores over that period of time (see Fig. 2a–c and Supplemental Table 3). In the overall population, metabolites from the TRP pathway were associated with changes in HRSD_17_ scores. 5HT, 5HIAA and the serotonin turnover marker 5HIAA/5HT showed significant positive and negative associations, respectively, with decreases over time in HRSD_17_ scores (FDR-adjusted *p* values <0.01).

We further subcategorized the population based on their HRSD_17_ scores after 8 weeks of treatment. If they had at least a 50% reduction in their HRSD_17_ scores, from baseline to exit, they were categorized as responders, otherwise they were nonresponders. We examined whether the temporal associations between metabolite changes and HRSD_17_ scores significantly differed between responders and nonresponders. The mean (±sd) HRSD_17_ scores in the responders and nonresponders were 21.86 (±5.17) and 22.03 (±4.28), respectively, at baseline, 10.10 (±5.77) and 15.03 (±6.58), respectively, at week 4, and 5.79 (±3.27) and 14.90 (±4.15), respectively, at week 8. 5HT temporal profiles significantly differed between the two groups, with the levels being consistently higher in the responders at baseline, week 4, and week 8, while the decline in HRSD_17_ scores was significantly lower at both 4 and 8 weeks compared with baseline (Fig. 2d, e). Levels of MHPG at baseline were significantly higher and the drop in MHPG levels over time was significantly greater in responders compared with nonresponders (Fig. 2f).

### Relationships amongst metabolites at baseline and after 8 weeks of treatment

**Fig. 3:**
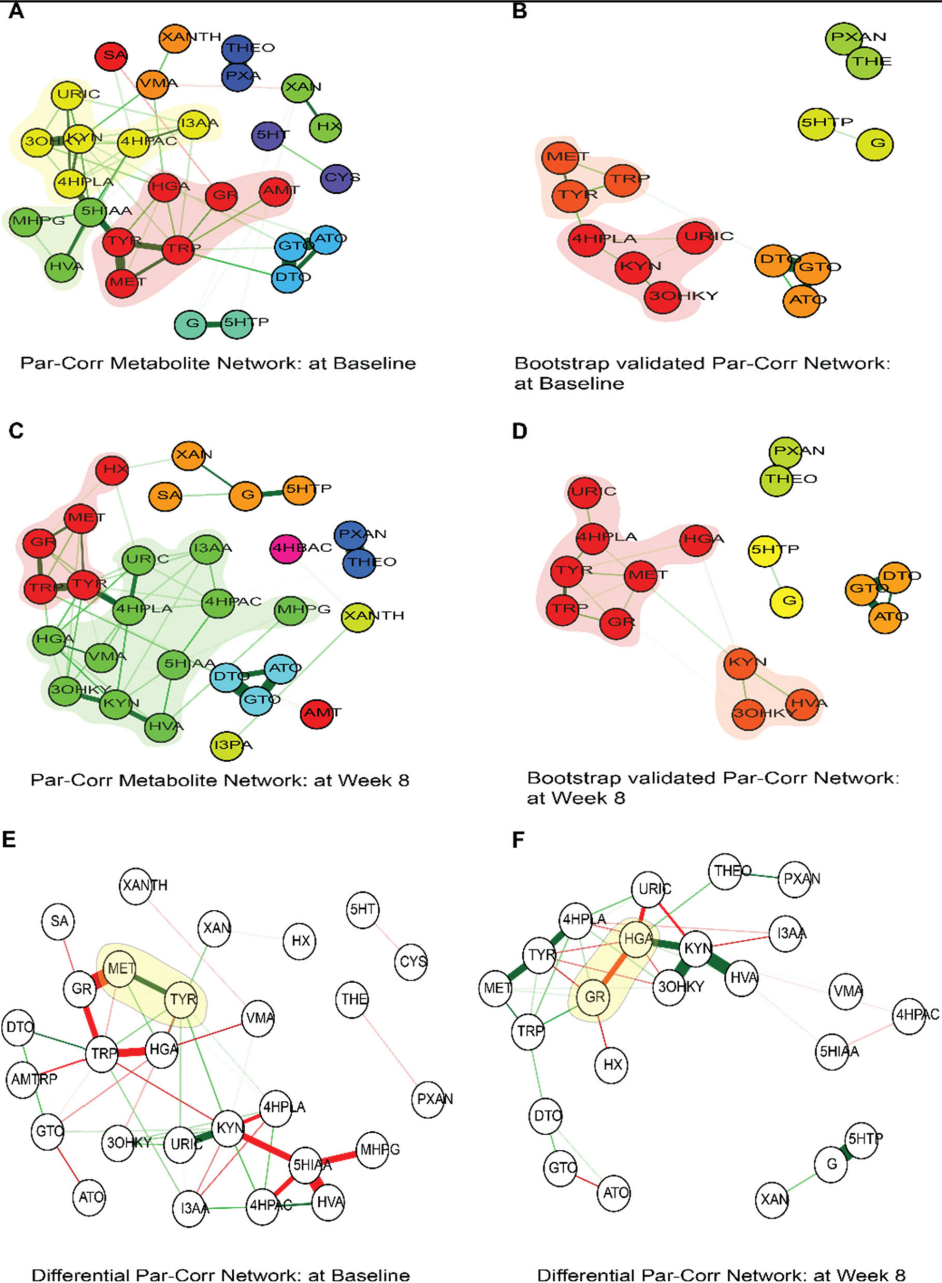
Partial correlation networks (PCN). **a** PCN at baseline; **b** PCN at baseline after 1000 bootstrap estimations; **c** PCN at week 8, and **d** PCN at week 8 after 1000 bootstrap estimations. The different clusters representing communities of closely associated metabolites are shown in different colors. Differential PCN as a function of high versus low HRSD_17_ week 8 scores at (**e**) baseline and (**f**) week 8. The edges between metabolites most impacted by higher HRSD_17_ week 8 scores are bolded in green, while those by lower HRSD_17_ week 8 scores are bolded in red

Biological systems are now increasingly viewed as complex networks of interlinked entities, topological analyses of which can reveal the underlying landscape of biological functionalities. Gaussian graphical modeling has been used to reconstruct pathway reactions in metabolomics data^57^. Combining a partial correlation network and genetic variation through GWAS has been shown to provide an in-depth overview of the underlying mechanistic pathways^58^. Here, using regularized partial correlation network analysis at baseline and also after week 8 of drug exposure (Fig. 3), we assessed the metabolite–metabolite interactions between tryptophan, tyrosine, purine, and tocopherol pathways.

Regularized partial correlation networks of the metabolites at baseline (Fig. 3a, b) and also at week 8 (Fig. 3c, d) showed significant correlations between several metabolites, both within and between pathways forming clusters of interacting molecules. A list of statistically significant partial correlations between metabolites at baseline and week 8 are presented in Supplemental Table 4 A, B. Important observations through cluster subgraph analysis showed that MET, TYR, and TRP formed a tight cluster both at baseline and week 8. However, the strength of interactions between MET and TYR was significantly reduced at week 8, compared with baseline (~50% reduction, permutation *p* value < 0.10). GR connection to this cluster was significant at week 8 through interactions with all three metabolites. HVA formed a significant correlation with KYN at week 8 that was not observed at baseline. Multiple other overlapping correlations in the two networks were observed at both baseline and week 8, suggesting that the majority of these interactions were a result of housekeeping biological interactions and were probably not entirely related to the drug effect.

### Differential partial correlation networks associated with HRSD_17_ scores at week 8

HRSD_17_ scores at week 8 indicated the depression status of the patients post drug treatment. We compared two partial correlation networks constructed with lower and higher values of HRSD_17_ scores at week 8 (the outcome status), using a median split, as a node. Our aim was to examine if the associations between metabolites were different between patients who responded to the drug better than those who responded poorly. Several metabolite–metabolite associations across the tyrosine, tryptophan, and purine pathways were found to be changed as a function of higher or lower outcome status (Supplemental Table 5 A, B). At baseline, GR–MET, TYR–MET, and KYN–URIC partial correlations were most impacted, while at week 8, KYN–HVA, KYN–3OHKY, 5HTP-G, and HGA–GR values were most impacted by HRSD_17_ week 8 status (Fig. 3e, f). Two sets of metabolite–metabolite interactions associated with the outcome status, HGA–GR interactions at week 8 and MET–TYR interactions at baseline, were further found to be statistically significant in linear regression models (highlighted in yellow in Fig. 3, e, f). The interaction plots based on linear regression models are presented in Supplemental Figs. 1 and 2. An interesting observation from the differential analysis of networks at baseline was that the partial correlations between metabolites that were differential between the low versus high HRSD_17_ networks involved several gut-microbe-related metabolites such as HGA, I3AA, 5HIAA, 4HPLA, and 4HPAC amongst others (Fig. 3e).

### Genetic influences on ratios of interacting metabolite pairs change during SSRI treatment

**Fig. 4:**
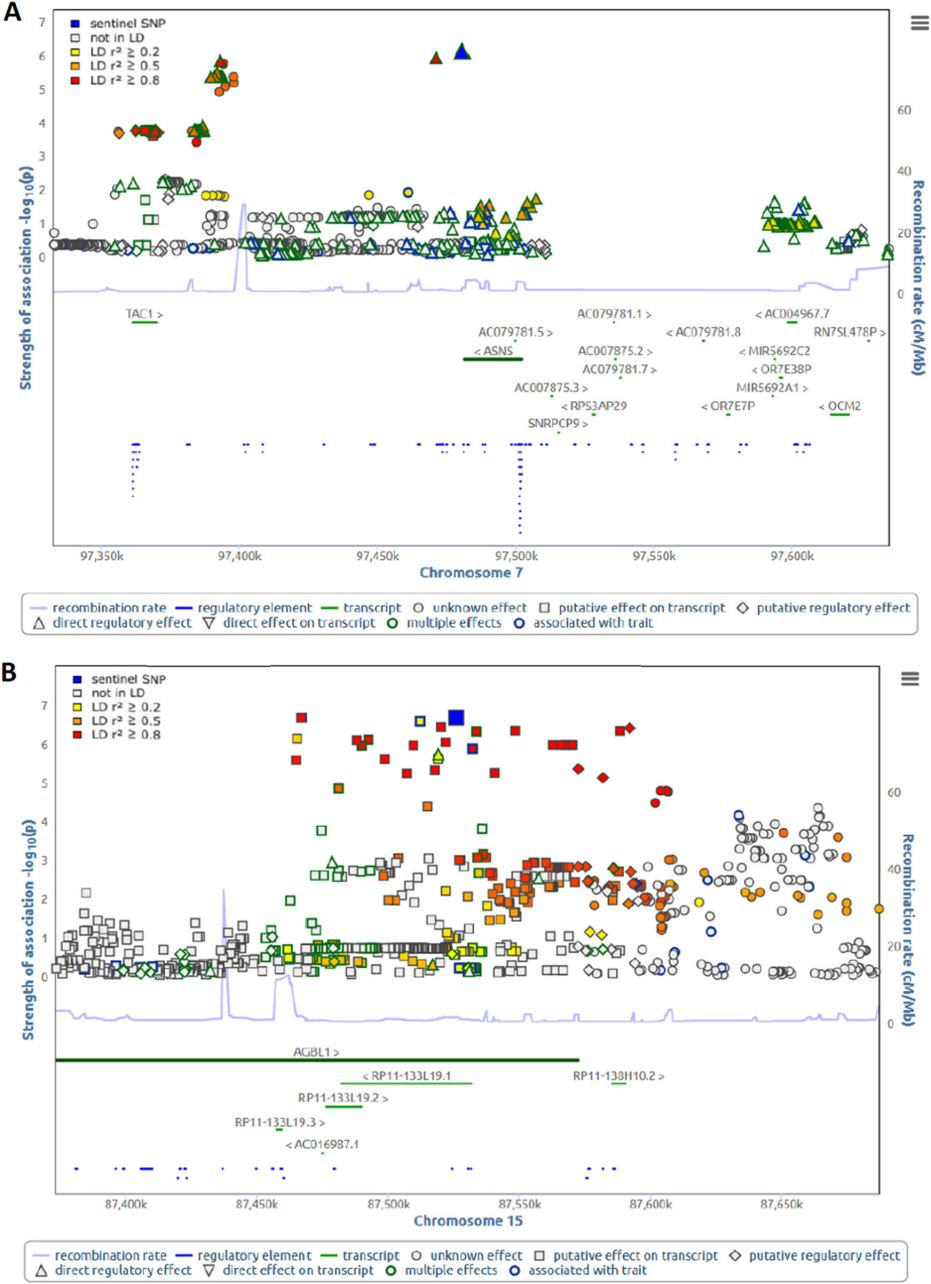
Plots showing regional association plots generated with SNiPA^61^ for: **a** the HGA/GR ratio at baseline and **b** the MET/TYR ratio at week 8

To identify potential modulators of significant metabolite–metabolite interactions and their differential interactions over time, we performed genome-wide association studies with the pairwise ratios of HGA/GR and MET/TYR in 288 subjects at each time point. To this end, we computed additive genetic associations of the two ratios with 5.55 mio autosomal SNPs at each time point, while adjusting for age, sex, time point-specific HRSD_17_ score, the first five PCs to account for population stratification. The strongest signal for the HGA/GR ratio was for rs55933921 on chromosome 7 (baseline: *P* = 8.59 × 10^−7^; week 4: *P* = 3.05 × 10^−3^; week 8: *P* = 1.14 × 10^−3^) in a locus spanning two genes, *TAC1* (protachykinin-1) and *ASNS* (asparagine synthetase [glutamine-hydrolyzing]). The strongest signal for the MET/TYR ratio was for rs2701431 on chromosome 15 (baseline: *P* = 5.57 × 10^−3^; week 4: *P* = 2.00 × 10^−4^; week 8: *P* = 8.48 × 10^−8^) in the *AGBL1* (ATP/GTP-binding protein like 1) locus (Fig. 4). Of note, genetic associations between these loci and metabolite ratios were the strongest at the time point that showed insignificant metabolite–metabolite interactions on the HRSD_17_ score.

## Discussion

We have applied a “targeted” electrochemistry-based metabolomics platform to quantitate the metabolomic profiles in MDD patients before and after SSRI treatment. Specifically, we assayed 31 neurotransmission-related metabolites that might have some relevance to MDD pathophysiology, based on published literature, including compounds from the tryptophan, tyrosine, and purine pathways, in plasma samples from 290 MDD patients at baseline, 4-, and 8 weeks of escitalopram/citalopram treatment. Metabolomic profiles were correlated with treatment response, defined as 50% reduction from baseline HRSD_17_ scores^.^ We found that plasma 5HT concentration was the most significantly decreased metabolite among all of the 31 metabolites after the drug treatment. Higher baseline 5HT levels were associated with better response to SSRI treatment. The 5HT levels in responders remained higher than that those in nonresponders all through the treatment period. Compared with the baseline metabolic state, significant shifts of metabolomic profiles to noncanonical branches of the three major pathways after drug exposure were noted, such as increase in the production of indoles in TRP metabolism, phenolic acids in phenylalanine/tyrosine metabolism, and the PXAN/XAN ratio in the purine pathway. In addition, changes in the catecholamine branch of tyrosine metabolism like increase in VMA and decrease in MHPG, and changes in the end products of purine pathway, such as decreases in XAN and HX, were identified in the MDD patients after drug exposure. Patients who had high MHPG at baseline responded better to SSRI treatment and their MHPG levels decreased more significantly than nonresponders. Temporal change in serotonin and 5HIAA significantly correlated with changes in HRSD_17_ scores over time. Partial correlation analysis between metabolites revealed that MET, TYR, and TRP formed a tight cluster of interacting molecules in these MDD patients. However, the strength of interactions (partial correlations) varied significantly pre- and post treatment. GR association with this cluster was significant at week 8. GWAS for the HGA/GR ratio identified two genetic loci that mapped to the TAC1 and ASNS genes, which are known to be involved in depression and neurotransmission, while the ratio MET/TYR identified the gene AGBL1 previously linked to neurodegeneration in mice.

Overall, significant perturbations within and between the tryptophan, tyrosine, and purine pathways due to the drug exposure, were noted. These findings are consistent with our previous metabolomic study of sertraline, another SSRI, in depressed patients^35^, where perturbations in TRP, in particular, changes in methoxyindole pathway and the ratio of KYN/TRP were correlated with treatment outcomes. Interestingly, plasma concentrations of the indoles synthesized from TRP, I3AA, and I3PA, were found to be significantly increased in the MDD patients in this study after the SSRI treatment. Plasma concentrations for I3AA and I3PA are known to be influenced by gut microbiota. Both I3AA and I3PA are aryl hydrocarbon receptor (AHR) agonists^59^, which could activate AHR transcriptional activity and modulate inflammation in the gut^60–63^ and brain^64,65^.

Indole-3-propionic acid is a potent hydroxyl radical scavenger produced exclusively by the commensal gut bacteria *Clostridium sporogenes*^66^ and normally found in the plasma and cerebrospinal fluid. I3AA, on the other hand, has been found to correlate significantly with both anxiety and depressive symptoms in chronic kidney disease patients (CKD)^67^. I3AA can be produced from indole by gut microflora^68^ in the intestines, or metabolized in tissues from tryptamine^69^ and other TRP derivatives. At uremic concentrations, I3AA has been linked to oxidative stress via AHR in CKD patients^70^. However, neither I3AA nor I3PA changes were correlated to changes in HRSD_17_ scores in our findings.

The most notable change in metabolic profiles after SSRI exposure occurred in the TRP metabolite, 5HT concentrations, which was expected from the mechanism of action of SSRI^38^. Plasma 5HT originates from the enterochromaffin cells in the gut and gets actively absorbed and stored by blood platelets, which highly express the 5HT transporter SLC6A4. SSRIs target SLC6A4 and inhibit 5HT uptake by platelets in blood. Therefore, a dramatic decrease in plasma 5HT concentration after the SSRI treatment can be expected in these patients. A higher concentration of plasma 5HT, which was stored in platelets, may reflect an elevated activity of the 5HT transporter, and, as a result, greater sensitivity to SSRIs in those patients. This hypothetical situation might explain why patients with higher plasma 5HT concentrations responded better to SSRIs.

Altered metabolic activity of the purine cycle has been linked with several MDD-related systemic responses, such as increased proinflammatory and oxidative processes^35^. The end products of purine metabolism, uric acid, a potent antioxidant, have been reported to be found in decreased levels in MDD^71–73^, while lower cerebro-spinal fluid (CSF) levels of hypoxanthine and xanthine, the two metabolites preceding uric acid, have previously been linked with depression^74^. Ali-Sisto et al., on the contrary, reported increased levels of xanthine to be associated with MDD^75^. In our study, we observed higher baseline levels of xanthine and hypoxanthine that decreased with the drug treatment. We did not detect increases in uric acid, but we did observe significant increases in the ratios of paraxanthine/xanthine and xanthosine/xanthine due to the drug exposure. This may indicate a potential beneficial effect of the drug through reducing oxidative stress by direct or indirect inhibition of the xanthine oxidase enzyme system. XO is known to generate vascular oxidative stress through reactive oxygen species production by catalyzing the hypoxanthine → xanthine → urate synthesis^76^. On the other hand, we observed increased associations between uric acid with 4HPLA and also with HGA at week 8 compared with baseline. Uric acid is known to function as an antioxidant (primarily in plasma) and pro-oxidant (primarily within the cell)^77^. Paraxanthine showed strong correlations to theophylline at all time points. It may also be possible that we were not able to detect significant increases in the levels of the antioxidant, uric acid, and the known psychostimulant paraxanthine^78^ due to their increased turnover rates brought about by the drug treatment.

In the tyrosine/phenylalanine pathway, 4HPAC and 4HBAC, the phenolic acid metabolites, were found to be significantly increased in MDD patients after SSRI treatment. Although these can potentially come from diets rich in plant-based foods, evidence suggests that these compounds can be produced through microbial fermentation of aromatic amino acids (AAAs) in the colon^79^. Although changes in the concentration of those metabolites were not associated with SSRI response in MDD patients, those changes possibly indicate alterations in gut microbiome or gut metabolism after citalopram/escitalopram treatment. 4HBAC is known for its antioxidant properties, as effective scavengers of free radicals and reactive nitrogen species, such as peroxynitrite^80^. 4HPAC is also known for its antioxidant, antiinflammatory, and anticancer activities^79^.

The strong interactions between MET–TYR–TRP, observed through partial correlation networks at baseline confirms the connection between folate-mediated methionine formation, leading to methyl donation reactions that form the monoamine neurotransmitters serotonin, dopamine, and epinephrine^81,82^. In depression, this balance is known to be perturbed^83^. With the drug exposure, we see further alterations in this balance at week 8. In addition, we see that GR significantly correlates with MET and TRP at week 8 post treatment. This may be indicative of changes in methylation status of the serotonin transporter^84^ through epigenetic mechanisms, in response to the SSRI treatment in these depression patients. At week 8, KYN–3OHKY association decreased significantly with concomitant increases in associations between KYN and the dopamine degradation product, HVA, and this association was comparitively stronger in patients who did not respond well to the treatment.

Using the ratios of metabolites significantly interacting as intermediate phenotypes leads us to rediscover loci known to be involved in neurotransmission/depression and neurodegeneration. The strongest association signals for baseline GR/HGA were within a locus on chromosome 7 containing two central genes: TAC1 (protachykinin-1) that has been linked to depression and anxiety^85^ and ASNS (asparagine synthetase [glutamine-hydrolyzing]) that is an important enzyme, the deficiency of which leads to substantial neurodevelopmental deficits^86^. Interestingly, patients with this deficiency (it is an inborn error of metabolism) also show modest changes in neurotransmitters. The strongest signal for MET/TYR was within a locus on chromosome 15 containing the gene AGBL1 (ATP/GTP binding protein-like 1) that has a role in controlling the length of the polyglutamate side chains on tubulin. This process is critical for neuronal survival, and the lack of such control has been reported to result in neurodegeneration in mice^87^. These findings underscores the utility of our “*Pharmacometabolomics-Informs- Pharmacogenomics*” approach^33^ to identify candidate genes for further functional studies. Using this strategy, we have previously identified SNP signals in the *DEFB1* and *AHR* genes that were associated with severity of depressive symptoms in these MDD patients^28^. DEFB1 is an antimicrobial peptide which is highly expressed and active in the gut^88^, playing a potentially important role in maintaining gut–microbiome homeostasis^89^. These results fit within the broadening body of information in support of important roles for the “microbiota–gut–brain axis” and inflammation in MDD pathophysiology.

Several limitations of this study warrant consideration. Compared with other MDD patients recruited in the PGRN-AMPS trial, study participants were “selected” because they were able to complete all three visits (i.e., baseline, 4, and 8 weeks) and provide blood samples, which would reduce the number of patients in the final sample who did poorly. In addition, only Caucasians were included in this study, and thus, a given inherent limitation was developed from analyzing a subset of MDD patients. Furthermore, the LCECA platform captures information on only redox-active compounds in the tyrosine, tryptophan, purine, and sulfur amino acid pathways and several markers of vitamin status and oxidative processes. The integration of data from lipidomics and mass spectrometry-based metabolomics platforms in future studies, as well as inclusion of several confounding variables, such as body mass index, diet, and lifestyle factors, would definitely help to better unravel the mechanistic aspect of the drug response.

In conclusion, we analyzed the metabolomic profile in 290 MDD patients before and after citalopram/escitalopram treatment. Noncanonical metabolic pathways related to TRP, tyrosine, and purine metabolism were found to be activated after the drug exposure. There was crosstalk among these pathways at baseline depression levels, which was significantly impacted by the drug exposure. Significant increases in gut–microbiota-related metabolites, such as the indoles and the phenolic acids, were observed in the overall population. Patients who responded to the drug compared to those who did not, had significant differences in baseline levels as well as in the trajectories of several metabolites, including several gut–microbiota related metabolites, suggesting that the drug exposure might be impacting gut-microbial ecology differently in the two groups. Overall, amelioration of oxidative stress and increases in anti-inflammatory processes seem to be part of the mechanism involved in response to citalopram/escitalopram treatment.

## Supplementary information

Supplemental material
