# Supplementary material for: Metabolomic signature of exposure and response to citalopram/escitalopram in depressed outpatients
Source: Transl Psychiatry. 2019 Jul 4;9:173. doi: 10.1038/s41398-019-0507-5 (PMC6609722; doi:10.1038/s41398-019-0507-5)
Supplement: Supplementary file 1 — Supplemental material [file 41398_2019_507_MOESM1_ESM.docx]

**Supplemental Tables and Figures**

**Table 1: Demographics and clinical features of study sample**

| **Variables** | **Total (N=290)** |
| --- | --- |
| **Age** | 39.8 (13.1)^a^ |
| **Gender** |  |
| F | 191 (65.9%)^b^ |
| M | 99 (34.1%)^b^ |
| **Treatment** |  |
| Citalopram | 120 (41.4%)^b^ |
| Escitalopram | 170 (58.6%)^b^ |
| **Response to Drug** | |
| No | 89 (30.7%)^b^ |
| Yes | 201 (69.3%)^b^ |
| **HRSD_17_** |  |
| Baseline | 21.9 (4.9)^a^ |
| W4 | 11.6 (6.4)^a^ |
| W8 | 8.6 (5.5^)a^ |

^a^ Mean (std dev); ^b^ Count (Percentage)

**Table 2 A): Significantly changing metabolites at unadjusted p<0.05 at week 4 and/or week 8.**

| Pathway | Metabolites | Fold Change^a^ | | Linear mixed model | | | |
| --- | --- | --- | --- | --- | --- | --- | --- |
|  |  |  |  | BL^b^ vs Week4 | | BL vs Week8 | |
|  |  | Week4/BL | Week8/BL | *p*-value | Adj *p*-value | *p*-value | Adj *p*-value^c^ |
| 1C+redox | CYS | -0.15 | -0.05 | 0.0488 | 1 | 0.4959 | 1 |
| 1C+redox | MET | -0.1 | 0.02 | 0.1922 | 1 | 0.7995 | 1 |
| Purine | G | -0.01 | -0.14 | 0.8894 | 1 | 0.0498 | 1 |
| Purine | GR | -0.12 | -0.11 | 0.1413 | 1 | 0.1714 | 1 |
| Purine | HX | -0.19 | -0.25 | 0.0106 | 0.575 | 0.0001 | 0.004 |
| Purine | PXAN | 0.03 | 0.15 | 0.6199 | 1 | 0.0034 | 0.183 |
| Purine | URIC | -0.01 | 0.02 | 0.7759 | 1 | 0.654 | 1 |
| Purine | XAN | -0.04 | -0.24 | 0.6068 | 1 | 0.0016 | 0.089 |
| Purine | XANTH | 0.08 | 0.21 | 0.4077 | 1 | 0.0088 | 0.473 |
| Purine | G_by_GR | 0.01 | -0.12 | 0.8847 | 1 | 0.0898 | 1 |
| Purine | XAN_by_G | 0 | 0.07 | 0.9773 | 1 | 0.3327 | 1 |
| Purine | GR_by_XANTH | -0.11 | -0.24 | 0.2353 | 1 | 0.0035 | 0.191 |
| Purine | XAN_by_XANTH | -0.09 | -0.31 | 0.3345 | 1 | 0.0002 | 0.013 |
| Purine | PXAN_by_XANTH | -0.03 | -0.03 | 0.6503 | 1 | 0.6374 | 1 |
| Purine | PXAN_by_XAN | 0.04 | 0.24 | 0.4796 | 1 | 0 | 0.002 |
| Purine | URIC_by_XAN | 0.03 | 0.24 | 0.6675 | 1 | 0.0017 | 0.092 |
| Purine | HX_by_XAN | -0.17 | -0.12 | 0.023 | 1 | 0.0704 | 1 |
| Tocopherol | ATOCO | 0.08 | 0.14 | 0.3296 | 1 | 0.0617 | 1 |
| Tocopherol | DTOCO | -0.02 | 0.09 | 0.755 | 1 | 0.2212 | 1 |
| Tocopherol | GTOCO.A | 0.05 | 0.17 | 0.523 | 1 | 0.0246 | 1 |
| Tryptophan | 3OHKY | -0.12 | 0.08 | 0.0497 | 1 | 0.1318 | 1 |
| Tryptophan | 3OHKY.b | 0.19 | 0.12 | 0.0127 | 0.685 | 0.0972 | 1 |
| Tryptophan | 5HIAA | -0.1 | 0.03 | 0.1164 | 1 | 0.5725 | 1 |
| Tryptophan | 5HT | -1.24 | -1.28 | 0 | 0 | 0 | 0 |
| Tryptophan | 5HTP | -0.05 | -0.18 | 0.5109 | 1 | 0.0229 | 1 |
| Tryptophan | I3AA | 0.14 | 0.18 | 0.0108 | 0.581 | 0.0004 | 0.024 |
| Tryptophan | KYN | -0.05 | 0.05 | 0.332 | 1 | 0.4253 | 1 |
| Tryptophan | TRP | -0.07 | 0.07 | 0.3114 | 1 | 0.3155 | 1 |
| Tryptophan | I3AA_by_TRP | 0.17 | 0.15 | 0.0007 | 0.038 | 0.0029 | 0.159 |
| Tryptophan | 5HTP_by_TRP | -0.04 | -0.19 | 0.5655 | 1 | 0.0191 | 1 |
| Tryptophan | KYN_by_TRP | 0 | 0 | 0.9384 | 1 | 0.9743 | 1 |
| Tryptophan | KYN_by_3OHKY | 0.1 | -0.05 | 0.1345 | 1 | 0.4609 | 1 |
| Tryptophan | 5HIAA_by_TRP | -0.06 | 0 | 0.3155 | 1 | 0.9979 | 1 |
| Tryptophan | 5HIAA_by_5HTP | 0.03 | 0.19 | 0.6782 | 1 | 0.0181 | 0.979 |
| Tryptophan | 5HT_by_5HTP | 0.61 | 0.52 | 0 | 0 | 0 | 0 |
| Tryptophan | 5HIAA_by_5HT | 1.15 | 1.23 | 0 | 0 | 0 | 0 |
| Tyrosine | 4HPAC | 0.22 | 0.31 | 0.0001 | 0.005 | 0 | 0 |
| Tyrosine | 4HPLA | -0.03 | 0.07 | 0.4622 | 1 | 0.0903 | 1 |
| Tyrosine | HGA | 0.11 | 0.15 | 0.1103 | 1 | 0.0399 | 1 |
| Tyrosine | HVA | -0.04 | -0.09 | 0.4513 | 1 | 0.2355 | 1 |
| Tyrosine | MHPG | -0.42 | -0.48 | 0 | 0 | 0 | 0 |
| Tyrosine | TYR | -0.03 | 0.08 | 0.5948 | 1 | 0.2033 | 1 |
| Tyrosine | VMA | -0.07 | 0.25 | 0.3867 | 1 | 0.001 | 0.055 |
| Tyrosine | HVA_by_TYR | -0.03 | -0.12 | 0.6612 | 1 | 0.1055 | 1 |
| Tyrosine | HGA_by_TYR | 0.13 | 0.13 | 0.0609 | 1 | 0.0709 | 1 |
| Tyrosine | 4HPAC_by_TYR | 0.24 | 0.27 | 0 | 0.001 | 0 | 0 |
| Tyrosine | MHPG_by_TYR | -0.31 | -0.43 | 0 | 0.001 | 0 | 0 |
| Tyrosine | VMA_by_TYR | -0.06 | 0.23 | 0.4592 | 1 | 0.0035 | 0.187 |
| Phe/Try | 4HBAC | 0.14 | 0.3 | 0.0534 | 1 | 0.0001 | 0.007 |
| Other | AMTRP | -0.09 | 0.05 | 0.215 | 1 | 0.4271 | 1 |
| Other | I3PA | 0.07 | 0.13 | 0.206 | 1 | 0.0194 | 1 |
| Other | SA | 0.05 | 0.15 | 0.3594 | 1 | 0.0171 | 0.921 |
| Other | THEOPHYLINE | -0.08 | 0.05 | 0.1805 | 1 | 0.3335 | 1 |

**Table 2 B): Descriptive statistics and linear mixed model results on the 31 metabolites***

|  | Mean(SD) | | | Fold Change | | Linear mixed model results - Week 4 | | | Linear mixed model results - Week 8 | | |
| --- | --- | --- | --- | --- | --- | --- | --- | --- | --- | --- | --- |
| Metabolites | BL | W4 | W8 | BL_W4 FC | BL_W8 FC | W4 Coef Est (Conf. Int) | W4 p | W4 Adjusted-p | W8 Coef Est (Conf. Int) | W8 p | W8 Adjusted-p |
| 3OHKY | 0.02 (0.94) | -0.1 (1.14) | 0.1 (0.91) | -0.12 | 0.08 | -0.1 (-0.2, -0.0) | 0.04973438 | 1 | 0.1 (-0.0, 0.2) | 0.131769 | 1 |
| 4HBAC | -0.13 (1) | 0 (0.91) | 0.16 (0.98) | 0.14 | 0.3 | 0.1 (-0.0, 0.3) | 0.05340039 | 1 | 0.3 (0.1, 0.4) | 0.0001324 | 0.12803 |
| 4HPAC | -0.18 (0.96) | 0.04 (0.99) | 0.13 (0.99) | 0.22 | 0.31 | 0.2 (0.1, 0.3) | 0.00010128 | 0.097938 | 0.3 (0.2, 0.4) | 8.71E-08 | 8.42E-05 |
| 4HPLA | -0.03 (0.98) | -0.06 (0.99) | 0.05 (0.99) | -0.03 | 0.07 | -0.0 (-0.1, 0.1) | 0.462213652 | 1 | 0.1 (-0.0, 0.2) | 0.09026237 | 1 |
| 5HIAA | 0.04 (1.06) | -0.06 (0.98) | 0.07 (0.98) | -0.1 | 0.03 | -0.1 (-0.2, 0.0) | 0.116414685 | 1 | 0.0 (-0.1, 0.2) | 0.57250907 | 1 |
| 5HT | 0.82 (0.97) | -0.42 (0.72) | -0.46 (0.72) | -1.24 | -1.28 | -1.2 (-1.3, -1.1) | 1.02E-62 | 9.88E-60 | -1.3 (-1.4, -1.2) | 1.67E-64 | 1.62E-61 |
| 5HTP | 0.09 (0.98) | 0.04 (1.02) | -0.09 (0.98) | -0.05 | -0.18 | -0.0 (-0.2, 0.1) | 0.5109039 | 1 | -0.2 (-0.3, -0.0) | 0.02286017 | 1 |
| AMTRP | 0.03 (1.02) | -0.06 (0.99) | 0.09 (0.95) | -0.09 | 0.05 | -0.1 (-0.2, 0.1) | 0.215047877 | 1 | 0.1 (-0.1, 0.2) | 0.42711271 | 1 |
| ATOCO | -0.06 (1.06) | 0.02 (1.03) | 0.08 (0.92) | 0.08 | 0.14 | 0.1 (-0.1, 0.2) | 0.329621719 | 1 | 0.1 (-0.0, 0.3) | 0.0617443 | 1 |
| CYS | 0.07 (0.75) | -0.08 (1.08) | 0.03 (0.96) | -0.15 | -0.05 | -0.2 (-0.3, -0.0) | 0.048802522 | 1 | -0.0 (-0.2, 0.1) | 0.49590243 | 1 |
| DTOCO | -0.01 (1.03) | -0.04 (1.02) | 0.07 (0.92) | -0.02 | 0.09 | -0.0 (-0.2, 0.1) | 0.755037825 | 1 | 0.1 (-0.1, 0.2) | 0.22119862 | 1 |
| GTOCO.A | -0.07 (1.05) | -0.02 (1.05) | 0.1  (0.9) | 0.05 | 0.17 | 0.1 (-0.1, 0.2) | 0.523045184 | 1 | 0.2 (0.0, 0.3) | 0.0245997 | 1 |
| HGA | -0.08 (1.05) | 0.02 (0.94) | 0.06 (1.06) | 0.11 | 0.15 | 0.1 (-0.0, 0.2) | 0.11026985 | 1 | 0.1 (0.0, 0.3) | 0.03985925 | 1 |
| HVA | 0.06 (0.76) | 0.02 (0.97) | -0.03 (1.21) | -0.04 | -0.09 | -0.0 (-0.2, 0.1) | 0.451285376 | 1 | -0.1 (-0.2, 0.1) | 0.23545053 | 1 |
| HX | 0.15 (0.85) | -0.04 (1.21) | -0.1 (0.95) | -0.19 | -0.25 | -0.2 (-0.3, -0.0) | 0.010649287 | 1 | -0.2 (-0.4, -0.1) | 8.12E-05 | 0.07848 |
| I3AA | -0.1 (1.01) | 0.04 (1.03) | 0.08 (0.94) | 0.14 | 0.18 | 0.1 (0.0, 0.2) | 0.010750683 | 1 | 0.2 (0.1, 0.3) | 0.00043768 | 0.42323 |
| I3PA | -0.04 (0.97) | 0.03 (0.96) | 0.09 (0.97) | 0.07 | 0.13 | 0.1 (-0.0, 0.2) | 0.20595762 | 1 | 0.1 (0.0, 0.2) | 0.01937234 | 1 |
| KYN | 0.01 (0.89) | -0.04 (0.92) | 0.06 (1.16) | -0.05 | 0.05 | -0.0 (-0.1, 0.0) | 0.331986513 | 1 | 0.1 (-0.1, 0.2) | 0.42530716 | 1 |
| MET | 0.04 (0.97) | -0.07 (1) | 0.05 (1.05) | -0.1 | 0.02 | -0.1 (-0.3, 0.1) | 0.192244839 | 1 | 0.0 (-0.1, 0.2) | 0.79946845 | 1 |
| MHPG | 0.28 (0.98) | -0.14 (1.02) | -0.19 (0.96) | -0.42 | -0.48 | -0.4 (-0.6, -0.3) | 6.52E-09 | 6.31E-06 | -0.5 (-0.6, -0.4) | 3.53E-13 | 3.41E-10 |
| SA | -0.07 (1) | -0.02 (0.92) | 0.08 (1.08) | 0.05 | 0.15 | 0.1 (-0.1, 0.2) | 0.359371952 | 1 | 0.1 (0.0, 0.3) | 0.01706288 | 1 |
| THEOPHYLINE | 0.02 (0.88) | -0.05 (1.09) | 0.08 (0.94) | -0.08 | 0.05 | -0.1 (-0.2, 0.0) | 0.180453024 | 1 | 0.1 (-0.1, 0.2) | 0.33351376 | 1 |
| TRP | 0 (1.02) | -0.07 (0.98) | 0.07 (1.02) | -0.07 | 0.07 | -0.1 (-0.2, 0.1) | 0.311358058 | 1 | 0.1 (-0.1, 0.2) | 0.31547711 | 1 |
| TYR | -0.02 (1.05) | -0.05 (0.97) | 0.06 (1) | -0.03 | 0.08 | -0.0 (-0.2, 0.1) | 0.594750892 | 1 | 0.1 (-0.0, 0.2) | 0.20331549 | 1 |
| URIC | -0.01 (0.95) | -0.02 (1.05) | 0.01 (1) | -0.01 | 0.02 | -0.0 (-0.1, 0.1) | 0.775885084 | 1 | 0.0 (-0.1, 0.1) | 0.65397172 | 1 |
| VMA | -0.07 (1.01) | -0.14 (1.12) | 0.18 (0.86) | -0.07 | 0.25 | -0.1 (-0.2, 0.1) | 0.386675434 | 1 | 0.2 (0.1, 0.4) | 0.00101521 | 0.98171 |
| XAN | 0.1 (0.97) | 0.06 (1.06) | -0.15 (0.98) | -0.04 | -0.24 | -0.0 (-0.2, 0.1) | 0.606808352 | 1 | -0.2 (-0.4, -0.1) | 0.00164188 | 1 |
| XANTH | -0.09 (1.29) | -0.01 (0.95) | 0.13 (0.61) | 0.08 | 0.21 | 0.1 (-0.1, 0.3) | 0.407704025 | 1 | 0.2 (0.1, 0.4) | 0.00875223 | 1 |
| G | 0.05 (0.99) | 0.04 (0.98) | -0.1 (1.04) | -0.01 | -0.14 | -0.0 (-0.1, 0.1) | 0.889392941 | 1 | -0.1 (-0.3, -0.0) | 0.04979412 | 1 |
| GR | 0.07 (1.3) | -0.04 (0.79) | -0.04 (0.84) | -0.12 | -0.11 | -0.1 (-0.3, 0.0) | 0.141293016 | 1 | -0.1 (-0.3, 0.0) | 0.1713917 | 1 |
| PXAN | -0.05 (0.99) | -0.03 (1.06) | 0.1 (0.88) | 0.03 | 0.15 | 0.0 (-0.1, 0.1) | 0.619938499 | 1 | 0.2 (0.1, 0.3) | 0.00339243 | 1 |

*All data are in the logarithmic space, standardized to unit variance

**Table 3: Metabolites significantly associated with changes in HRSD**_17_ **score over time**.

| Pathway | Metabolites | Coef Estimate (95% CI) | P-value | Adjusted P-value |
| --- | --- | --- | --- | --- |
| Purine | GR/XANTH | 0.457 (0.043, 0.871) | 0.03 | 1 |
| Tryptophan | 3OHKY | -0.560 (-0.981, -0.139) | 0.009 | 0.431 |
| Tryptophan | 5HIAA | -0.819 (-1.268, -0.370) | <0.001 | 0.017 |
| Tryptophan | 5HT | 1.724 (1.246, 2.202) | <0.001 | <0.001 |
| Tryptophan | 5HTP | 0.442 (0.020, 0.864) | 0.04 | 1 |
| Tryptophan | 5HTP/TRP | 0.452 (0.027, 0.876) | 0.037 | 1 |
| Tryptophan | 5HIAA/TRP | -0.733 (-1.198, -0.269) | 0.002 | 0.095 |
| Tryptophan | 5HIAA/5HTP | -0.567 (-0.986, -0.148) | 0.008 | 0.379 |
| Tryptophan | 5HIAA/5HT | -1.998 (-2.486, -1.511) | <0.001 | <0.001 |
| Tyrosine | HGA | -0.519 (-0.943, -0.094) | 0.017 | 0.792 |
| Tyrosine | VMA | -0.491 (-0.906, -0.077) | 0.02 | 0.954 |
| Tyrosine | HGA/TYR | -0.526 (-0.952, -0.099) | 0.016 | 0.742 |

**Table 4:**

**A). Significant Partial Correlations between Metabolites at Baseline**

| pcor | node1 | node2 | pval | qval | prob |
| --- | --- | --- | --- | --- | --- |
| 0.675121 | PXAN | THEOPHYLINE | 2.22E-16 | 3.42E-14 | 1 |
| 0.497369 | DTOCO | GTOCO.A | 2.22E-16 | 3.42E-14 | 1 |
| 0.441644 | ATOCO | GTOCO.A | 2.22E-16 | 3.42E-14 | 1 |
| 0.356224 | 3OHKY | KYN | 7.74E-11 | 8.92E-09 | 1 |
| 0.350343 | G | 5HTP | 1.65E-10 | 1.51E-08 | 1 |
| 0.347158 | MET | TYR | 2.47E-10 | 1.90E-08 | 0.999733 |
| 0.272889 | 4HPLA | TYR | 8.98E-07 | 5.49E-05 | 0.999733 |
| 0.270203 | TRP | TYR | 1.16E-06 | 6.68E-05 | 0.999633 |
| 0.264603 | ATOCO | DTOCO | 1.95E-06 | 0.0001 | 0.998614 |
| 0.254313 | HX | XAN | 4.96E-06 | 0.000229 | 0.992514 |
| 0.237321 | MET | TRP | 2.12E-05 | 0.000889 | 0.959223 |
| 0.206711 | GR | TRP | 0.000224 | 0.007769 | 0.959223 |
| 0.206346 | 5HIAA | HVA | 0.00023 | 0.007935 | 0.959223 |
| 0.203251 | I3AA | 4HPAC | 0.000286 | 0.009436 | 0.90876 |
| 0.192854 | XANTH | VMA | 0.000586 | 0.017419 | 0.90876 |
| 0.187816 | TRP | AMTRP | 0.000818 | 0.022612 | 0.90876 |
| 0.186702 | KYN | 4HPLA | 0.000879 | 0.023872 | 0.816552 |
| 0.177562 | GR | 4HBAC | 0.001573 | 0.038717 | 0.816552 |

**B) Significant Partial Correlations between Metabolites at week 8**

| pcor | node1 | node2 | pval | qval | prob |
| --- | --- | --- | --- | --- | --- |
| 0.658993 | PXAN | THEOPHYLINE | 2.22E-16 | 5.14E-14 | 1 |
| 0.464725 | DTOCO | GTOCO.A | 2.22E-16 | 5.14E-14 | 1 |
| 0..40203 | ATOCO | GTOCO.A | 4.97E-14 | 7.68E-12 | 0.999999 |
| 0.320313 | G | 5HTP | 3.58E-09 | 3.14E-07 | 0.999999 |
| 0.319619 | TRP | TYR | 3.89E-09 | 3.29E-07 | 0.999999 |
| 0.317972 | ATOCO | DTOCO | 4.71E-09 | 3.64E-07 | 0.999903 |
| 0.282116 | KYN | HVA | 2.39E-07 | 1.55E-05 | 0.999903 |
| 0.276417 | 3OHKY | KYN | 4.24E-07 | 2.46E-05 | 0.998525 |
| 0.245298 | G | XAN | 7.86E-06 | 0.000352 | 0.998525 |
| 0.244773 | 4HPLA | TYR | 8.23E-06 | 0.000365 | 0.998525 |
| 0.242564 | URIC | 4HPLA | 9.98E-06 | 0.00042 | 0.995347 |
| 0.234402 | GR | TRP | 2.00E-05 | 0.000773 | 0.974866 |
| 0.218193 | TRP | AMTRP | 7.43E-05 | 0.002647 | 0.925041 |
| 0.191281 | DTOCO | TRP | 0.000533 | 0.015595 | 0.925041 |
| 0.190841 | MET | TYR | 0.000549 | 0.01597 | 0.925041 |
| 0.185741 | URIC | HGA | 0.000775 | 0.020724 | 0.925041 |
| 0.183455 | MET | GR | 0.000902 | 0.023072 | 0.925041 |
| 0.181866 | KYN | 4HPLA | 0.001002 | 0.02477 | 0.925041 |
| 0.180506 | G | AMTRP | 0.001094 | 0.026261 | 0.925041 |
| 0.178186 | XANTH | I3PA | 0.001271 | 0.028869 | 0.925041 |
| 0.177028 | HX | XAN | 0.001369 | 0.030194 | 0.589758 |

**Table 5**

**A) Partial Correlations between metabolites at baseline that were differential as a function of HRSD_17_ week8 scores (high and low).**

| **Var1** | **Var2** | **value** | **abs.value** |
| --- | --- | --- | --- |
| GR | MET | -0.21363 | 0.213626 |
| HVA | 5HIAA | -0.18833 | 0.188327 |
| HGA | TRP | -0.13828 | 0.138283 |
| KYN | URIC | 0.131126 | 0.131126 |
| MHPG | 5HIAA | -0.12369 | 0.12369 |
| KYN | 5HIAA | -0.12237 | 0.12237 |
| TRP | GR | -0.11792 | 0.117924 |
| TYR | MET | 0.109504 | 0.109504 |
| 4HPAC | 5HIAA | -0.10584 | 0.105837 |
| 4HPLA | KYN | -0.09449 | 0.094495 |
| HVA | 4HPAC | 0.078584 | 0.078584 |
| AMTRP | TRP | -0.0724 | 0.0724 |
| TRP | DTOCO | 0.070782 | 0.070782 |
| VMA | HGA | -0.0682 | 0.068195 |
| GTOCO.A | ATOCO | -0.05956 | 0.059555 |
| TYR | HGA | -0.05508 | 0.055079 |
| TRP | KYN | -0.05388 | 0.053881 |
| 4HPAC | I3AA | 0.052862 | 0.052862 |
| 4HPAC | KYN | 0.049198 | 0.049198 |
| GTOCO.A | DTOCO | 0.04834 | 0.04834 |
| TYR | KYN | 0.045176 | 0.045176 |
| 4HPLA | I3AA | -0.04459 | 0.044586 |
| 4HPLA | 4HPAC | 0.043339 | 0.043339 |
| 3OHKY | URIC | 0.043281 | 0.043281 |
| SA | GR | -0.04069 | 0.040686 |
| KYN | I3AA | -0.03557 | 0.035569 |
| HGA | MET | 0.035145 | 0.035145 |
| TYR | TRP | 0.034156 | 0.034156 |
| KYN | 3OHKY | 0.03188 | 0.03188 |
| HGA | XAN | 0.03153 | 0.03153 |
| HGA | GTOCO.A | -0.03094 | 0.030938 |
| TRP | I3AA | 0.029927 | 0.029927 |
| X4HPLA | URIC | -0.02931 | 0.029307 |
| TYR | URIC | 0.026114 | 0.026114 |
| 4HPLA | 3OHKY | 0.023646 | 0.023646 |
| TRP | MET | -0.02218 | 0.022184 |
| HGA | 3OHKY | -0.02204 | 0.022039 |
| KYN | GTOCO.A | 0.016984 | 0.016984 |
| THEOPHYLINE | PXAN | -0.01658 | 0.016583 |
| 5HT | CYS | -0.01542 | 0.015422 |
| VMA | XANTH | -0.01386 | 0.013862 |
| TYR | 4HPLA | 0.011252 | 0.011252 |
| VMA | KYN | 0.006332 | 0.006332 |
| TRP | GTOCO.A | -0.0059 | 0.005896 |
| XAN | HX | 0.005493 | 0.005493 |
| TYR | 3OHKY | 0.004615 | 0.004615 |
| 4HPLA | MET | -0.00348 | 0.003476 |
| I3AA | URIC | -0.00247 | 0.00247 |
| DTOCO | ATOCO | -0.00168 | 0.001679 |
| AMTRP | G | -0.00049 | 0.00049 |
| 5HTP | G | -0.00031 | 0.000308 |

**B) Partial Correlations between metabolites at week8 that were differential as a function of HRSD_17_ week8 scores (high and low).**

| **Var1** | **Var2** | **value** | **abs.value** |
| --- | --- | --- | --- |
| 5HTP | G | 0.268378 | 0.268378 |
| HVA | KYN | 0.233725 | 0.233725 |
| KYN | 3OHKY | 0.231145 | 0.231145 |
| HGA | KYN | 0.174382 | 0.174382 |
| TYR | MET | 0.173171 | 0.173171 |
| TYR | 4HPLA | 0.16872 | 0.16872 |
| HGA | GR | -0.15729 | 0.157291 |
| HGA | URIC | -0.14155 | 0.141546 |
| KYN | URIC | -0.12069 | 0.120692 |
| THEOPHYLINE | PXAN | 0.109611 | 0.109611 |
| TRP | MET | 0.107477 | 0.107477 |
| GTOCO.A | ATOCO | -0.1011 | 0.101102 |
| HX | GR | -0.0996 | 0.099601 |
| TRP | GR | 0.094342 | 0.094342 |
| KYN | I3AA | -0.08513 | 0.085125 |
| TYR | GR | -0.08429 | 0.084286 |
| 4HPLA | URIC | 0.066794 | 0.066794 |
| GTOCO.A | DTOCO | 0.065136 | 0.065136 |
| THEOPHYLINE | HGA | 0.064168 | 0.064168 |
| TYR | HGA | -0.0634 | 0.063397 |
| XAN | G | 0.063155 | 0.063155 |
| HGA | 3OHKY | -0.06165 | 0.061647 |
| 4HPLA | KYN | -0.06112 | 0.061121 |
| 4HPLA | TRP | 0.055847 | 0.055847 |
| TYR | 3OHKY | -0.0557 | 0.055703 |
| TRP | DTOCO | 0.054064 | 0.054064 |
| 4HPLA | 3OHKY | 0.038043 | 0.038043 |
| 4HPLA | GR | 0.034975 | 0.034975 |
| 4HPLA | I3AA | -0.03056 | 0.030556 |
| TYR | TRP | 0.028623 | 0.028623 |
| 4HPAC | 5HIAA | -0.02668 | 0.026678 |
| HVA | HGA | 0.022111 | 0.022111 |
| DTOCO | ATOCO | 0.021298 | 0.021298 |
| GR | MET | 0.0137 | 0.0137 |
| HVA | 5HIAA | 0.011931 | 0.011931 |
| 3OHKY | MET | 0.01152 | 0.01152 |
| 4HPLA | 4HPAC | -0.01107 | 0.011073 |
| TYR | URIC | 0.011021 | 0.011021 |
| VMA | 4HPLA | 0.00405 | 0.00405 |
| KYN | 5HIAA | -0.0028 | 0.002799 |
| VMA | HGA | -0.00148 | 0.001477 |

**Supplemental figures 1 and 2**

| 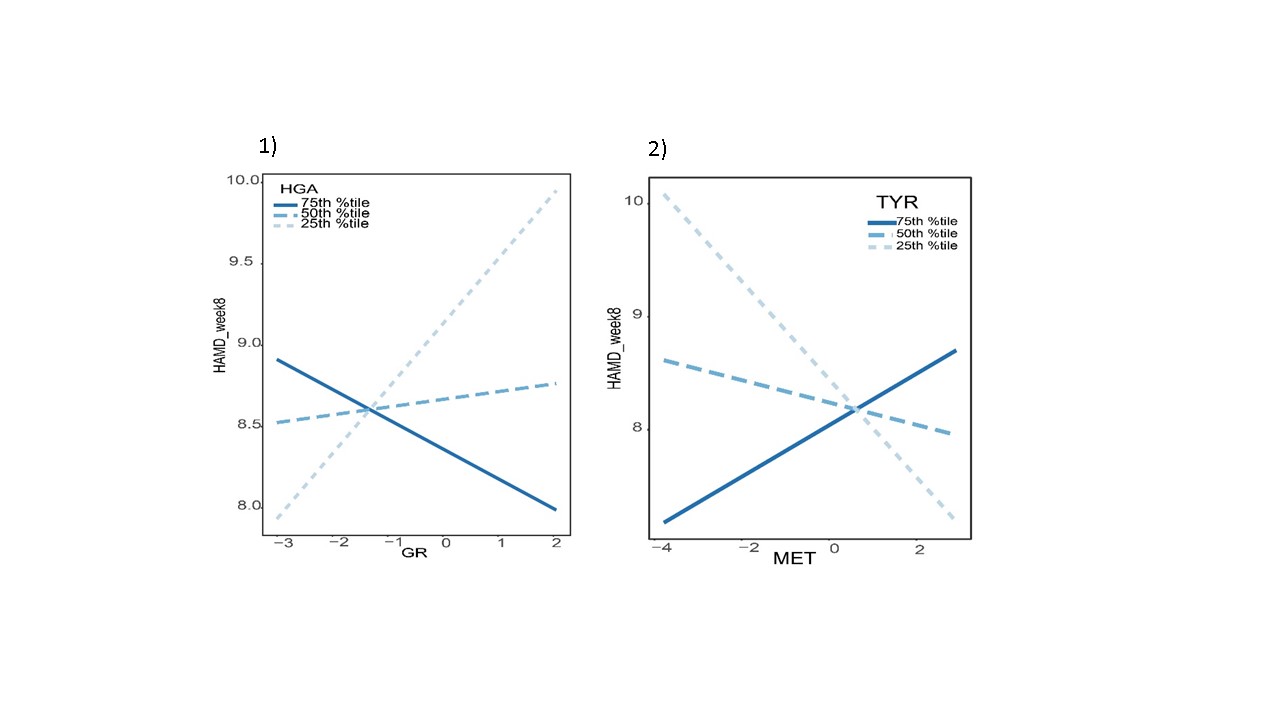 |
| --- |

**FlowChart for Data Analysis**

| **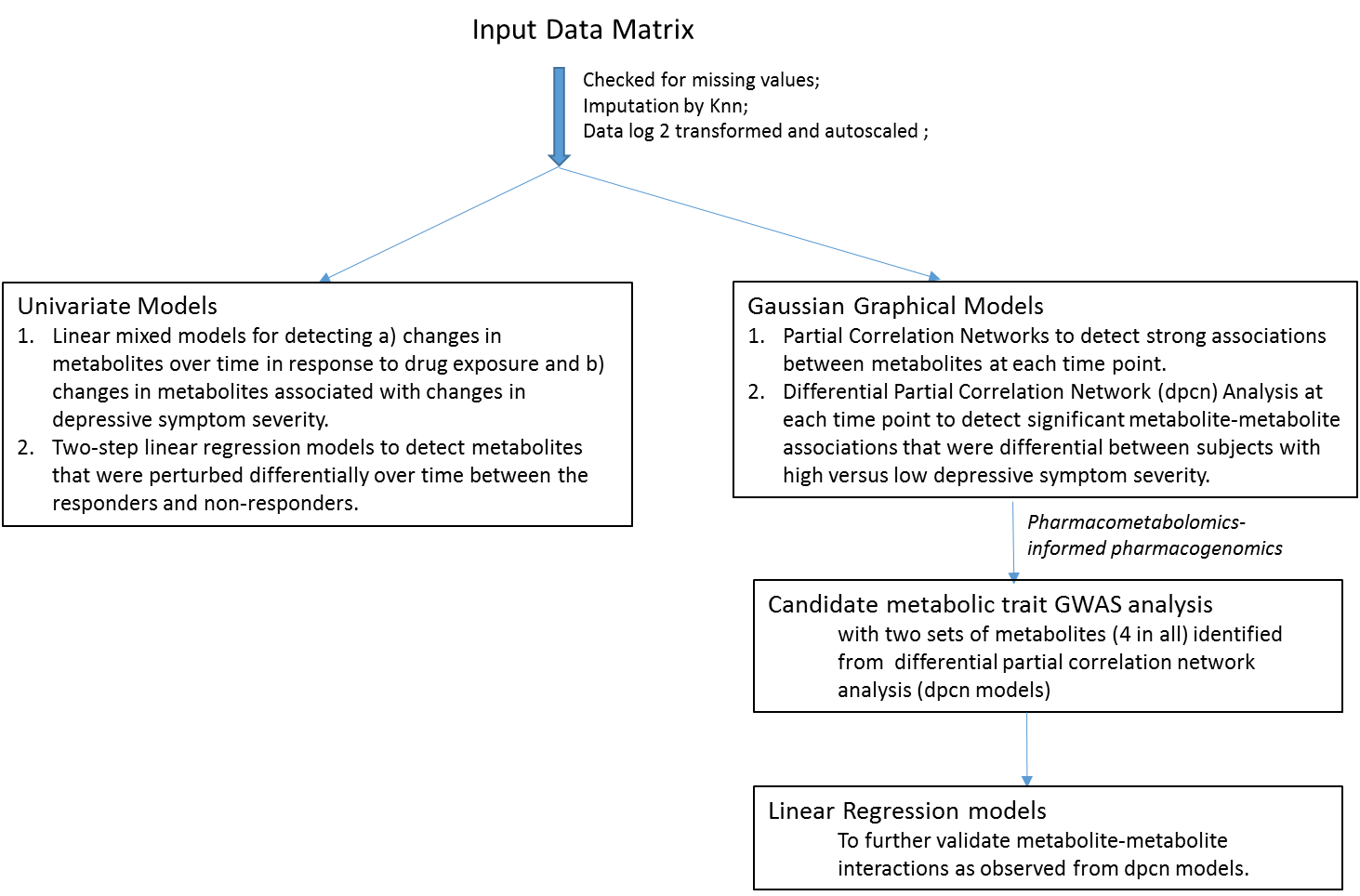** |
| --- |

**Boxplots for 31 metabolites**

| **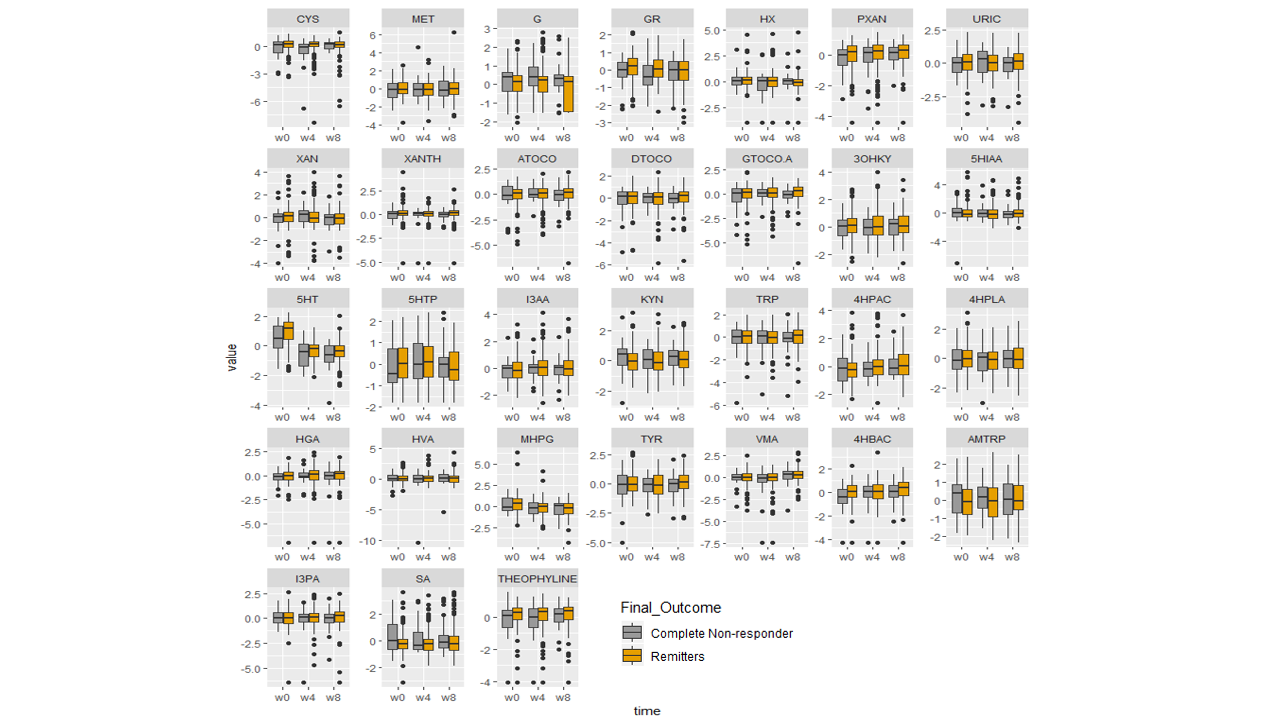** |
| --- |
